# A comparison of phylogenetic and distance-based approaches for the distinction of genetically closed species,* Draba rimarum* (Rech.f.) A.R. Khosravi & A. Eslami-Farouji, and *Draba aucheri *Boiss. (Brassicaceae) as a case study

**DOI:** 10.22099/mbrc.2023.47706.1842

**Published:** 2023

**Authors:** Atena Eslami-Farouji, Ahmad Reza Khosravi, Mahdi Gholami, Sasan Mohsenzadeh

**Affiliations:** Department of Biology, School of Science, Shiraz University, Shiraz, Iran

**Keywords:** Cruciferae, Distance-based methods, Draba, Genetically close species

## Abstract

Circumscribing species boundries is necessary in systematic plant biology. Even a mistake in delimiting taxa may lead to incorrect scientific interpretations. *Draba rimarum *(Rech.f.) A.R. Khosravi & A. Eslami-Farouji is an endemic Iranian species with a narrow geographic distribution, and is genetically close to *D. aucheri*. The present study provided a phylogenetic review, time divergence, and planar network of both species to unravel the distinct position of both species along with the prediction of any conflicting or ambiguous signals. Regarding this purpose, here we represent that phylogenetic trees may fail to show reliable results toward the distinct position of genetically close species.

## INTRODUCTION

Genomic DNA sequence is significantly dedicated to clarification of enigmatic issues within the taxonomy, plant systematics, population genetics, evolutionary biology, and ecology [e.g., 1]. Although molecular data remarkably increase our understanding of the phylogenetic relationships among taxa, these data may fail to provide a practical solution to circumscribe species boundaries [e.g., 2]. In contrast with those solely interested in molecular data [e.g., 3], almost all researchers interested in combine molecular results with morphological supplementary data to shed further light on answer scientific questions and prevent confusion [e.g., 4-8, etc.]. Based on Tripp and Lendemer [9], researchers should preliminary pay attention to non-molecular characters, and only if these traits would not informative enough, then molecular datasets will be helpful. However, some authors ignored morphological characters [10] and directly used molecular databases to examine evolutionary relationships among taxa. Closely related species may be hard to distinguish [e.g., 11], and in the case of newly diverged species, genetic distance is not sufficiently accumulated. According to Tripp and Lendemer [9] statement, the divergence calculation of the closely related species is significantly suggested. Moreover, the presence of recombination, hybridization, conflicting and ambiguous signals are evolutionary processes that make the phylogenetic trees hard to follow [12, and references therein]. Practically, phylogenetic approaches gain limited evolutionary information, and authors erroneously believed that a single gene does not reflect the whole genome results (for more examples see [13]). Evolutionary reconstruction analysis shows a lower level of error (2-8%), while matrix distance methods observed an error of 9 to 15% [13]. Nevertheless, sufficient variation needs for the former method [13 and references therein]. 

The most challenging scenario is to use an alternative algorithm to show even a very low genetic distance between species. A distance-matrix approach explores the actual evolutionary distance between studied groups by converting heterogeneity into splits [14]. Algorithms that compute planar split networks seem to be applicable here and including split decomposition [14], median networks [15], and Neighbor-Net [16]. 

The validity of the above-mentioned scenarios has been discussed by different workers. Split decomposition is a distance-based method [17], and is a helpful approach to show evolutionary distance or dissimilarities between closely related taxa [14], especially when the number of studied species is small. In other words, in the case of small or very similar data, split decomposition gains much better-resolved graphs than those of neighbor-net networks [18]. In contrast to the network derived by the Neighbor-Net approach, split decomposition networks contain all available splits [19]. The generation of splits is dissimilar in split decomposition and Neighbor-Net approaches [20]. Gauthier and Lapointe [21] proposed to use a split decomposition approach other than a median network method. Based on Bandelt and Dress [14] statement, the trustability of the method has already been proved. The Neighbor-Net (distance-based) [19] methodology is characterized by less conservation, and referred to use for large datasets [22]. The median-joining algorithm is mainly based upon phenetics (distance-based) and low genetic distance [23]. 

To the reader's knowledge, our study inspired by species that inspite of morphological differences are genetically related (e.g., *Hesperis ilamica* A. Eslami-Farouji, Khodayari & Assadi and *H. straussii* Bornm. in [24] and *Draba rimarum* (Rech.f.) A.R. Khosravi & A. Eslami-Farouji and *D. aucheri* Boiss. in [25]. In the present study, owing to the shortcomings of phylogenetic approach, we are looking for an approach that help us in distinguishing species with low genetic distances, and find possible conflicting signals (e.g., hybridization, recombination, etc.) within the studied taxa.

## MATERIALS AND METHODS

**Taxon Sampling: **Regarding the phylogenetic study, almost all sequences used for the molecular study were obtained from NCBI. Sequence identities were carefully tested via BLAST (The Basic Local Alignment Search Tool). We were critically focused on *D. rimarum* and closely related species (*D. aucheri*, *D. incompta* Steven, and *D. pulchella* Willd. ex DC.) to find enough evidence for our work. Indeed, we were aware that the higher number of the studied accessions for each species, will increase the reliability of the results, therefore, we performed field studies to sample more individuals for each taxon. Nonetheless, owing to the scarce and geographically restricted distribution and patchy habitat of the taxa, we only sampled and sequenced one accession regarding the phylogenetic analyses. However, nine samples of *D. rimarum*, and eleven specimens of *D. aucheri* were morphologically investigated in our recent work [25]. Above-mentioned samples are belonging to different herbaria, and the voucher information of HSHU (Herbarium of Shiraz University) palnt materials are available in [25] and Online Resource 1. Overall, to resolve the true taxonomic position of *Draba rimarum* and relatives, a set of 24 accessions of *Draba *L. (20 species, annuals and perennials) plus two genera (*Arabis *L. and* Pseudoturritis* Al-Shehbaz, 7 species) as outgroups, were selected to perform phylogenetic analyses (Supplementary Table S1). Outgroups are selected based on Jordon-Thaden et al. [26] investigation. Details about the studied specimens are summarized in Online Resource 1. Based on numerous studies performed by previous workers [see 25 and references therein], It does not make sense to add all *Draba* or *Arabis* specimens in our analyses, and we only selected a limited number of species herein.

**Molecular Study: **DNA extractions, PCR amplifications, sequencing and phylogenetic analyses were directly followed in the research carried out by Khosravi et al. [8]. We know the significance of using different molecular markers to find more robust results. However, in the case of genetically closed species (Table S1), less conservative markers (e.g., nrDNA) sound to be more helpful than those of chloroplastic ones. 

**Phylogenetic Network: **Low genetic distances existing within the studied species make researchers reconstruct phylogenetic networks [15]. Almost all authorities have stated that these networks are effective tools for handling the true evolutionary ancestor-descendant relationships within the studied data and the profound understanding of that [e.g., 27-29]. 

Using the split-decomposition approach, a phylogenetic network analysis was performed [14, 30] via SplitsTree4 ver.4.11.3 [18, 31] within ITS dataset for detecting putative evolutionary relationship within the studied *Draba*. The network is rooted by *D. olympica *Sibth. ex. DC., *D. lasiocarpa* Rochel, *D. acaulis* Boiss., *D. cretica* Boiss. & Heldr. and *D. hispanica* Boiss,. This split graph clearly outlines the evolutionary distances within studied taxa [32]; Likewise, the level of the reticulation signals was calculated by delta scores [33] in SplisTree for each species. Neighbor-Net [31, 34] and Median-Joining split graphs were also regenerated by SplitsTree4 ver.4.11.3. The first approach, along with 2000 replication bootstraps, was constructed by the GTR model to test the network trustworthiness, while the second approach was performed by the equal site rate variation and the Median-Joining (MJ) model character transformation. Our general strategy was to define the standard default parameters for the remaining values. 

**Divergence Time Estimations: **DTE (divergence time estimation) was measured by a secondary calibration approach for measuring the radiation time of the most recent common ancestor (TMRCA) of *D. rimarum* and its closely related species (e.g.,* D. aucheri* and *D. pulchella*) through BEAST v.1.10 [35] on CIPRES science gateway [http://www.phylo.org/; 36]. According to the information available in Couvreur et al., [37] the calibration point was executed at a mean of 16.8 (95% HPD, 10.0–3.48) Ma. Based on what [38] mentioned, the divergence time estimation done by different approaches [37, 39-41] seems to be similar; thus, we decided to follow Couvreur et al. [37]. Empirical base frequencies, Gamma plus invariant sites for site heterogeneity model, and lognormal uncorrelated relaxed clock type [42] were chosen for constructing DTE analyses. The SYM+I+G model, based on Akaike information criterion [43], was applied as the most appropriate evolutionary model to the ITS. We also used the Speciation: Yule process as tree priors and a descriptor of diversification events [44]. Finally, the xml file was created and Markov chain Monte Carlo (MCMC) runs, sampling every 3000 generations, were implemented for 30 million generations. The results were examined in Tracer v.1.6 [45]. Effective sample size (ESS) represented reliable statistics (ESS>200), and proved the convergence of runs within 30 million generations for nrDNA. 

## RESULTS

**Figure 1:**
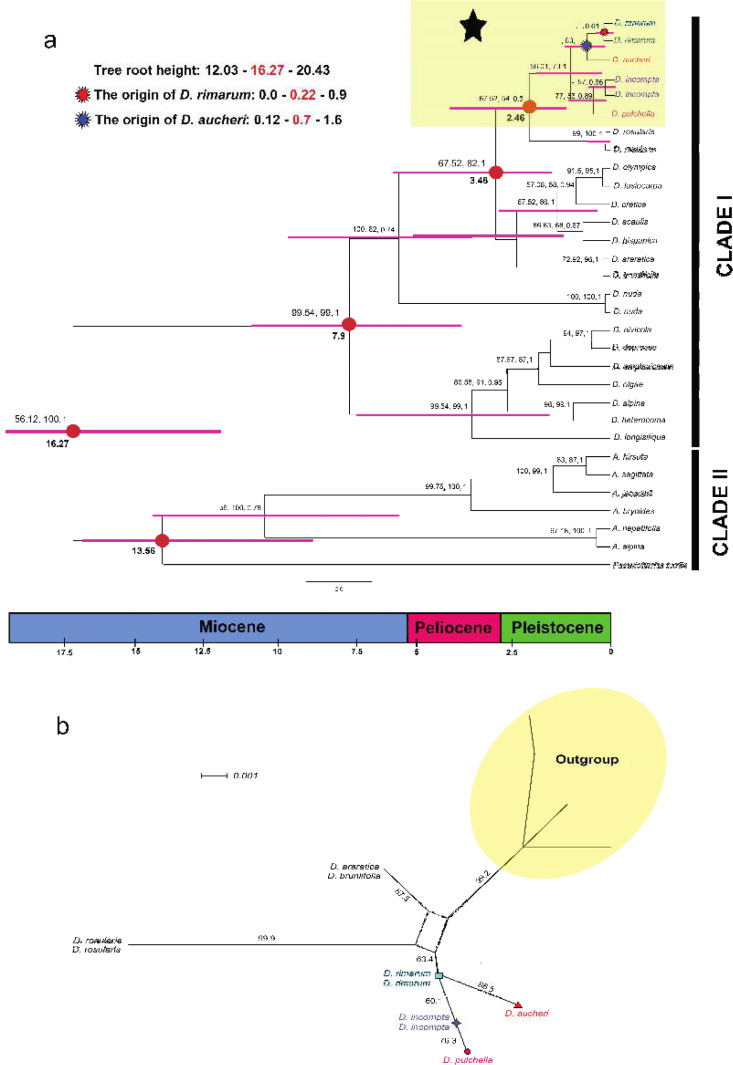
**a)** Bayesian molecular dated tree of studied *Draba* species inferred by BEAST based on nrDNA dataset and a secondary calibration point in Iran. The calibration point comprises 16.27 Ma and mainly focused on *D. rimarum* and closely related species. Pink bars show the 95% HPD (highest posterior density) intervals around the average ages in each single node. Tree root height along with *D. rimarum* (red star) and *D. aucheri* (blue star) origin are also represented. Numbers above each taxon refer to the clade credibility (PP> 50) **b)** Split-decomposition graph based on ITS data of studied *Draba* species using SplitTree software. Scale bar demonstrates split-decomposition distance of 0.01. Numbers on each edge corresponds to bootstrap values (BS> 50, 2000 replicates). Parallelograms represent conflicting splits. The colors in a and b sections are in agreement with Fig. 1 in [25]

The Bayesian tree was constructed based on the ITS sequences of 31 accessions (27 species). The final alignment contains 592 characters, of which 451 are constant, 446 are conserved, 141 are variable sites, and 88 are potentially parsimony-informative. The maximum parsimony analyses yielded a tree with a length of 327, consistency index (CI) of 0.52 and retention index (RI) of 0.59. The evolutionary tree containing BI, MP and ML branch supports is shown in Figure 1a. 

The evolutionary tree comprises two well-supported monophyletic clades, designated I and II. Each clade has been subdivided into subclades. Clade credibility values (PP) are shown in Fig. 1a. The main idea of this passage belongs to *D. rimarum* and its closely related species. Thus, we decided to focus only on CLADE I, subclade I, which is marked with a black asterisk and is highlighted with pale yellow color. In CLADE I, *D. rimarum* and *D. aucheri* are distinct from *D. incompta* and *D. pulchella*. *Draba rimarum* and *D. aucheri* lack strong branch support; as a result, due to high PP values, the monophyly of subclade I is moderately supported, while we can not obtain enough evidence to show the distinct position of *D. rimarum* and *D. aucheri*. 

The phylogenetic tree, owing to the high affinity of sequences within studied taxa, failed to unravel the true evolutionary relationship between *D. rimarum* and *D. aucheri*; thus, the non-tree-like graph is recommended herein (Fig. 1b). As we discussed before, the central topic of this research is *D. rimarum *and its closely related species. Therefore, we have attempted to construct the network with selected species from the phylogenetic tree (see Fig. 1a). This network is based on ITS dataset and is the first which recovered *D. rimarum* and its phylogenetically related species. All three networks (split decomposition, Neighbor-Net and Median-joining) demonstrate similar results; thus, we decided to depict the first graph as the most informative network herein. The split decomposition network [14, 30] for studied *Draba* represents a non-planar graph. The divergent clusters have substantially resolved the separation of the *Draba aucheri* from *D. rimarum*. The split network with a fit value of 83.24 comprises 14 splits in total, and leads to the split decomposition network of 19 vertices and 22 edges (Fig. 1b). The available boxes within the network might represent the possibility of recombination(s) [32] within the studied taxa (Fig. 1b); Nonetheless, this study did not find any evidence for recombination (p= 0.36) based on the phi test (pairwise homoplasy index; [46]). In condition that the greatest delta score belongs to *D. acaulis* (0.28) and *D. pulchella *(0.27), the lowest delta score is detected in *D. incompta* (0.19) and *D. aucheri* (0.21), respectively. The average delta score is also estimated to be 0.27.

The evolutionary tree obtained from BEAST is visualized in Fig. 1a. The present study represents the recent radiation of the studied species within *Draba *(Fig. 1a). *Draba rimarum* and *D. aucheri* were originated around 0.22 (95% HPD: 0-0.9 Ma; red star, Fig. 1a) and 0.7 (95% HPD: 0.12-1.6 Ma; blue star, Fig. 1a) Ma in the Pleistocene, respectively. The divergence of *D. pulchella* is also dated to the Pleistocene. 

## DISCUSSION

Authors [25] morphologically confirmed the distinct taxonomic identity of *D. rimarum *and *D. aucheri*. Darwin [47] stated that in contrast to widespread species, narrow endemics do not morphologically diversified. However, even genetically closed species (Table S1), may show sufficient morphological divergence [23]. Herein, we tried to establish a method to reassess [23] recent controversy, phylogenetically.

In the case of phylogenetic surveys, the monophyly of *Draba *has been proved by previous studies [e.g., 48-49]. Based on our analyses, the three *Draba *species (*D. aucheri*, *D. pulchella* and *D. incompta*) are sisters to *D. rimarum*. However, the statistical branch supports were not strongly confirmed by ITS analyses, and in our eyes, this phylogenetic tree (ITS) cannot resolve the true relationship between *D. aucheri* and *D. rimarum* (Fig. 1a). According to Bandelt et al. [15], phylogenetic reconstruction of individuals with small genetic distances is a complicated task. Nevertheless, Müller et al. [11] stated that ITS_2_ is a straightforward marker to distinguish closely related species, but we believed that in the case of considerable genetic similarity among taxa, phylogenetic analyses failed to do so. In this case, non-tree like networks are helpful to unravel true relationships with different number of datasets [e.g., 50-51]. Obviously, the first author of this paper (AEF) was faced with the same situation in her studies regarding two species of *Hesperis* L.: *Hesperis ilamica* and *H. straussii*. The Phylogenetic relationship of the both species were not clearly discovered, as they were recently diverged from each other [24]. 

Phylogenetic networks generalize the phylogenetic trees in a circular order and able to show numerous trees concurrently [52] with definite distances [12]. Split-decomposition [14] is a prominent non-treelike approach for reconstructing phylogenetic network [53], and examining the presence of recombination in SplitsTree [18, 32]; Indeed, split decomposition attempts to shed further light on the true phylogenetic relationship of studied group, and this algorithm perform well even after some levels of ambiguous signals are present [14]. Huson [54] stated that this is a conservative approach that performs better on small datasets and closely related taxa, and adequately efficient to estimate evolutionary distances (see Fig. 1b). The present study accepted Huson [54] regarding the trustworthy of this methology in small number of studied taxa (Fig. 1b). Thus, results represent the evolutionary relationships and possible ancestral connections in Iranian *Drabas* based on ITS (Fig. 1b). In particular, in the case of molecular characters, numerous specimens (≥10) of closely related taxa should be evolutionary analyzed [9]. However, in some cases, species are found to be narrow endemics, which are geographically confined to limited areas. Cosequently, only limited number of them are available for molecular and non-molecular surveys.

The molecular dating estimation indicates that the divergence time between *D. rimarum* and *D. aucheri* occurred around 0.12–1.6 (0.7 Ma) in the Pleistocene (Fig. 1a); Thus, it is clear that *D. rimarum* has diverged from its relatives recently in the Pleistocene. Beilstein and Windham [55] provided well-defined evidence about the recent divergence of north American *Draba* specimens. *Draba *is supposed to be radiated in the Pleistocene [26, 56]. We also propose Pleistocene radiation events for *D. rimarum*. Ecologically, the alpine regions, owing to the optimum precipitations that they have, most likely provided proper niches for plant species [38]. As a result, we assume that Iranian *Drabas* have critically preferred higher elevations. Though, the disjunct distribution centers of both *D. aucheri* and *D. rimarum* (see Fig. 1 in [25]) confirmed their independent evolution within the Pleistocene.

## Conflict of Interest:

Authors have a financial relationship with the organization that sponsored the research. 

## Supplementary materials
