# Supplementary material for: A comparison of phylogenetic and distance-based approaches for the distinction of genetically closed species, Draba rimarum (Rech.f.) A.R. Khosravi & A. Eslami-Farouji, and Draba aucheri Boiss. (Brassicaceae) as a case study
Source: Mol Biol Res Commun. 2023;12(4):155–63. doi: 10.22099/mbrc.2023.47706.1842 (PMC10599596; doi:10.22099/mbrc.2023.47706.1842)
Supplement: Supplementary file 1 [file mbrc-12-155-s001.pdf]

Table S1. Estimation of the evolutionary divergence between *Draba rimarum*, and genetically related species was conducted using Tamura et al. [57-58] methodology.

	Draba_nuda	Draba_cretica	Draba_acaulis	Draba_rosularis_a	Draba_rosularis_b	Draba_araratica	Draba_lasiocarpa	Draba_olympica	Draba_hispanica	Draba_bruniifolia	Draba_aucheri	Draba_pulchella	Draba_incompta_a	Draba_incompta_b	Draba_rimarum_a	Draba_rimarum_b
Draba_nuda																
Draba_cretica	0.0580189105															
Draba_acaulis	0.0561741545	0.0086454415														
Draba_rosularis_a	0.0600265398	0.0244457445	0.0226866902													
Draba_rosularis_b	0.0600265398	0.0244457445	0.0226866902	0.0000000000												
Draba_araratica	0.0467949236	0.0138388573	0.0121020445	0.0156166178	0.0156166178											
Draba_lasiocarpa	0.0522457441	0.0086129575	0.0103867890	0.0262228044	0.0262228044	0.0155870180										
Draba_olympica	0.0540951670	0.0103445889	0.0121285936	0.0279954279	0.0279954279	0.0173340364	0.0017106829									
Draba_hispanica	0.0522457441	0.0051587299	0.0034371557	0.0190898518	0.0190898518	0.0085968073	0.0068863872	0.0086155276								
Draba_bruniifolia	0.0467949236	0.0138388573	0.0121020445	0.0156166178	0.0156166178	0.0000000000	0.0155870180	0.0173340364	0.0085968073							
Draba_aucheri	0.0523339773	0.0156144574	0.0174001127	0.0191204399	0.0191204399	0.0121213009	0.0173340364	0.0190840727	0.0138430200	0.0121213009						
Draba_pulchella	0.0442363151	0.0178315758	0.0160077368	0.0196001070	0.0196001070	0.0124242783	0.0177676764	0.0195618856	0.0141887538	0.0124242783	0.0088244172					
Draba_incompta_a	0.0467164292	0.0138692621	0.0156213358	0.0173392743	0.0173392743	0.0103640934	0.0155622132	0.0173064055	0.0120828494	0.0103640934	0.0051490902	0.0035146286				
Draba_incompta_b	0.0467164292	0.0138692621	0.0156213358	0.0173392743	0.0173392743	0.0103640934	0.0155622132	0.0173064055	0.0120828494	0.0103640934	0.0051490902	0.0035146286	0.0000000000			
Draba_rimarum_a	0.0485563706	0.0121249462	0.0138734433	0.0155917180	0.0155917180	0.0086291688	0.0138210271	0.0155622132	0.0103476823	0.0086291688	0.0034297272	0.0052766605	0.0017106829	0.0017106829		
Draba_rimarum_b	0.0485563706	0.0121249462	0.0138734433	0.0155917180	0.0155917180	0.0086291688	0.0138210271	0.0155622132	0.0103476823	0.0086291688	0.0034297272	0.0052766605	0.0017106829	0.0017106829	0.0000000000	
